# Investigation of Novel Aronia Bioactive Fraction-Alginic Acid Nanocomplex on the Enhanced Modulation of Neuroinflammation and Inhibition of Aβ Aggregation

**DOI:** 10.3390/pharmaceutics17010013

**Published:** 2024-12-25

**Authors:** Bong-Keun Jang, Soo Jung Shin, Hyun Ha Park, Vijay Kumar, Yong Ho Park, Jeom-Yong Kim, Hye-Yeon Kang, Sunyoung Park, Youngsun Kwon, Sang-Eun Shin, Minho Moon, Beom-Jin Lee

**Affiliations:** 1 Department of Pharmacy, College of Pharmacy, Ajou University, Suwon 16499, Republic of Korea; salrigra@ajou.ac.kr; 2 JBKLAB, Inc., 17 Techno 4-ro, Yuseoung-gu, Daejeon 34013, Republic of Korea; joykim@cellmed.com (J.-Y.K.); khy@cellmed.com (H.-Y.K.); sunyoung@cellmed.com (S.P.); dudtjs134@cellmed.com (Y.K.); sn8120@cellmed.com (S.-E.S.); 3 Department of Biochemistry, College of Medicine, Konyang University, 158, Gwanjeodong-ro, Seo-gu, Daejeon 35365, Republic of Korea; tlstnzz@konyang.ac.kr (S.J.S.); 22801504@konyang.ac.kr (H.H.P.); vijay10187@konyang.ac.kr (V.K.); 22851609@konyang.ac.kr (Y.H.P.); 4 JBKLAB, Inc., 464 Dunchon-daero, Jungwon-gu, Seongnam-si 13229, Republic of Korea; 5 Research Institute for Dementia Science, Konyang University, 158, Gwanjeodong-ro, Seo-gu, Daejeon 35365, Republic of Korea; 6 Institute of Pharmaceutical Science and Technology, Ajou University, Suwon 16499, Republic of Korea

**Keywords:** anthocyanin, aronia bioactive fraction–alginic acid nanocomplex, electrostatic interaction, nanoparticles, enhanced stability, neuroinflammation, reduced aβ aggregation

## Abstract

Background/Objectives: Aronia extract or its active compounds, especially anthocyanin, have shown potential for Alzheimer’s disease (AD)-related pathologies, including neuroinflammation, fibrillogenesis of amyloid beta (Aβ), and cognitive impairment. However, there was still concern about their structural instability in vivo and in vitro. To solve the instability of anthocyanins, we combined aronia bioactive factions (ABFs) and alginic acid via electrostatic molecular interactions and created an ABF–alginic acid nanocomplex (AANCP). We evaluated whether it is more stable and effective in cognitive disorder mice and neuroinflammation cell models. Methods: The physicochemical properties of the AANCP, such as nanoparticle size, structural stability, and release rate, were characterized. The AANCP was administered to scopolamine-injected Balb/c mice, and to BV2 microglia treated with lipopolysaccharide (LPS) and amyloid beta (Aβ). Inflammation responses were measured via qPCR and ELISA in vitro, and cognitive functions were measured via behavior tests in vivo. Results: The AANCP readily formed nanoparticles, 209.6 nm in size, with a negatively charged zeta potential. The AANCP exhibited better stability in four plasma samples (human, dog, rat, and mouse) and was slowly released in different pH conditions (pH 2.0, 7.4, and 8.0) compared with non-complexedABF. In vitro studies on microglial cells treated with AANCPs revealed a suppression of inflammatory cytokines (tumor necrosis factor-alpha and interleukin-6) induced by LPS. The AANCP increased microglial Aβ phagocytosis through the activation of triggering receptor expressed on myeloid cell 2 (TREM2)-related microglial polarization. The AANCP inhibited aggregation of Aβ in vitro and alleviated cognitive impairment in a scopolamine-induced in vivo dementia mouse model. Conclusions: Our data indicate that AANCPs are more stable than ABFs and effective for cognitive disorders and neuroinflammation via modulation of M2 microglial polarization.

## 1. Introduction

Alzheimer’s disease (AD), which accounts for 60–80% of all dementia cases, is an irreversible neurodegenerative disease. Amyloid beta (Aβ), a pathological hallmark of AD, synergizes with neuroinflammation, thereby hastening disease progression [1,2,3]. In particular, Aβ pathology and pro-inflammatory microglia form a vicious cycle, contributing to the pathogenesis of AD [2,4]. Aβ aggregates directly activate microglia as they are recognized through pattern recognition receptors, such as scavenger receptors and Toll-like receptors, on the surface of microglia [5]. In addition, pro-inflammatory cytokines released by activated microglia not only induce neuronal damage but also exacerbate Aβ pathology by inducing upregulation of β-secretase 1 levels [6,7]. Therefore, disrupting this vicious cycle of Aβ pathology and neuroinflammation may be a potential target for the treatment of AD.

Neuroinflammation has emerged as an important target concerning AD, with various therapeutics being developed to alleviate AD-related pathologies by modulating neuroinflammation [8,9]. Microglia play opposing roles in neurodegeneration and neuroprotection [10,11,12], which are related to the functional phenotypes of microglia, M1 and M2. Microglial phenotypes are regulated by the pathological conditions of toxic factors, such as lipopolysaccharide (LPS) and Aβ [13,14]. The M1 phenotype secretes pro-inflammatory cytokines and induces neurotoxicity, synapse pruning, and demyelination in the brain [13,15,16]. Conversely, the M2 phenotype, which has anti-inflammatory functions, performs protective homeostatic functions such as phagocytosis and network remodeling [4]. In addition, the triggering receptor expressed on myeloid cell 2 (TREM2), a microglial surface receptor, is associated with microglial phenotype switching in neurological diseases such as AD, Parkinson’s disease, tauopathy, and ischemic stroke [17,18,19,20]. Moreover, TREM2 is known to be an essential receptor for Aβ recognition and phagocytosis in the AD brain [21,22]. Thus, promoting the conversion of M1 microglia to the M2 phenotype may be an important therapeutic strategy in AD.

Although several pharmaceutical compounds have been extensively investigated for their ability to alleviate AD pathogenesis, aducanumab and lecanemab are the sole medications sanctioned by the Food and Drug Administration (FDA) [23,24]. Despite the demonstrated reduction in amyloid plaques by aducanumab and lecanemab, monoclonal antibodies face serious challenges, such as high treatment costs and low therapeutic efficacy [25,26]. Therefore, several studies have explored the potential of natural compounds as therapeutics for AD [27,28,29]. Natural products offer low treatment costs and enhance the efficacy of AD by targeting various pathways [30,31]. Particularly, Aronia berries, also known as black chokeberries, are edible fruits that resemble cherries [32]. Aronia berries have gained significant attention because of their abundant polyphenol content, particularly anthocyanins, which can comprise up to 1.5% of their fresh weight [33]. The Aronia fruit extract reportedly exhibits anti-inflammatory effects [34,35,36,37], whereas the dry extract reduces the secretion of pro-inflammatory cytokines, such as TNF-α and IL-1β, in LPS-stimulated macrophages [35]. Additionally, the Aronia berry extract inhibits the activity of nuclear factor kappa-light-chain-enhancer of activated B cells (NF-κB) in LPS- and high-fat-induced inflammation in in vitro and in vivo models, respectively [38]. Anthocyanins, abundant in aronia, reportedly modulate microglial phenotypes, inhibit neuroinflammation, and suppress Aβ_40_-induced production of reactive oxygen species [39]. Furthermore, cyanidin-3-O-glucoside (C3G), a major component of anthocyanin, inhibits the fibrillogenesis of Aβ and degrades aggregated Aβ [40]. Although Aronia extract has the potential to serve as a disease-modifying drug targeting both neuroinflammation and Aβ pathology for the treatment of AD, anthocyanins are highly susceptible to chemical and enzymatic degradation via hydration reactions [41], which increase the excretion of anthocyanins, thereby reducing their intestinal absorption in vivo and inducing their degradation or loss of activity, resulting in low bioavailability and therapeutic efficacy. However, these anthocyanins can suppress degradation by forming ionic bonds with negatively charged polymers, such as fucoidan, resulting in stabilized nanocomplexes [42].

In this study, we prepared an ABF-alginic acid nanocomplex (AANCP) by chemically linking anthocyanins to negatively charged alginic acids. We predicted that AANCP would solely inhibit the hydration of anthocyanins while retaining its ability to mitigate AD pathology because AANCP is engineered to only form ionic bonds with alginic acid. The physicochemical properties, such as zeta potential, particle size, and surface morphology of AANCP, were characterized. The release rate and the stability of AANCP were also determined. Finally, the therapeutic effectiveness of AANCP was assessed, including the activation of the M1 and M2 phenotype, the conversion of the M1 microglial phenotype to the M2 phenotype for reducing inflammation and promoting tissue repair, the inhibition of the aggregation of Aβ, and the attenuation of cognitive decline in a scopolamine-induced dementia model.

## 2. Materials and Methods

### 2.1. Materials

The Aronia melanocarpa berries were purchased from Grennvit and Greenvit Co., Ltd. (Zambrow, Poland). The alginic acid was obtained from Qingdao Bright Moon Group Co., Ltd. (Qingdao, China). The Aβ_42_ peptide and 5-Carboxyfluorescein-labeled Aβ_1–42_ (FAM-Aβ) were purchased from Bachem AG (Bubendorf, Switzerland) and AnaSpec (Fremont, CA, USA), respectively. Aβ_42_ was dissolved in a 0.1 M aqueous ammonia solution, as previously described [43]. Thioflavin T (ThT), also known as Basic Yellow 1, was purchased from Tokyo Chemical Industry Co., Ltd. (Tokyo, Japan). Morin, 4, 6-diamidino-2-phenylindole Fluoroshield™ (DAPI), scopolamine hydrobromide trihydrate (scopolamine), and LPS and ionized calcium-binding adapter molecule 1 (Iba-1) antibody were obtained from Sigma-Aldrich (St. Louis, MO, USA) and Abcam (Cambridge, UK), respectively.

### 2.2. Preparation and Characterization of AANCP 

#### 2.2.1. Preparation of ABF and AANCP

The fruit of Aronia melanocarpa was extracted with 50% ethanol, and the extract was freezing-dried after filtering in a polyphenol adsorption resin. This process increased the content of cyanidin-3-glucoside (C3G), a type of anthocyanin, to 16%, which we named ABF. Alginic acid was broken down into low-molecular alginic acid by treatment of citric acid (pH 4.0). The ABF was combined with low-molecular alginic acid in deionized water (DW) as a ratio of 1:4 at room temperature for 48 h by generation of ABF-alginic acid nanocomplex (AANCP).

#### 2.2.2. Physical Characterization of AANCP

Fabricated AANCP were analyzed with dimension and zeta potential by dynamic light scattering (DLS) using a Malvern ZEN3600 Zetasizer and analyzed utilizing Zetasizer software version 7.11 (Malvern Panalytical, Malvern, Worcestershire, UK). The morphology of AANCP was observed using a Hitachi SU8230 field-emission scanning electron microscope (FE-SEM; Hitachi, Tokyo, Japan). Samples were prepared by depositing 10 μL of the AANCP solution onto a cover glass, which was allowed to dry completely for 24 h at room temperature.

### 2.3. Release Characteristics of AANCP

ABF (20 mg) and AANCP (100 mg) were dissolved in 5 mL of phosphate buffers of pH 2.0, 7.4, and 8.0 to obtain final concentrations of 4 and 20 mg/mL, respectively. Anthocyanin standard was detected to absorbance at 520 nm using a Varioskan LUX Multimode Microplate Reader (Thermo Fisher Scientific, Waltham, MA, USA). Anthocyanin release was tracked at multiple time points over 48 h, and the percentage of released anthocyanins was calculated to analyze the release kinetics under different pH conditions. 

### 2.4. Chemical Stability of ABFand AANCP in Plasmas

The ABF or AANCP were mixed with four different plasmas of human, dog, rat, and mouse, respectively, to obtain the final concentration of 10 μM of Cyanidin-3-O-galactoside (Cy3Gal). The mixtures were incubated for 120 min at 37 °C, and then samples were taken out at 0, 20, 40, 60, and 120 min, respectively. The chemical stabilities of ABF and AANCP were determined by quantitative analysis of Cy3Gal using 5500 HPLC-QTRAP-MS/MS system (AB SCIEX, Framingham, MA, USA).

### 2.5. Cell Culture and Viability of BV2 Microglial Cells

BV2 microglial cells were cultured in Dulbecco’s Modified Eagle Medium (DMEM) supplemented with 10% fetal bovine serum (FBS) and 100 units/mL penicillin-streptomycin (Gibco, Grand Island, NY, USA). The BV2 microglial cells were seeded at 5 × 10^3^ in a 96-well microplate and incubated for 24 h at 37 °C. Next, the cells were treated with ABF and AANCP concentrations of 100 to 800 μg/mL for 24 h at 37 °C. Subsequently, a solution of WST-1 (DoGenBio, Seoul, Republic of Korea) was added to each well, and the cells were incubated for 2 h. The cells were shaken for 1 min, and the absorbance was quantified at 450 nm using a SpectraMax iD3 multimode microplate reader with SoftMaxPro7.1 Setup Software (Molecular Devices, San Jose, CA, USA).

### 2.6. In Vitro Analysis of Pro-Inflammatory Cytokine Secretion from Microglia

The secretion of pro-inflammatory cytokines from BV2 microglial cells was measured using common ELISA kits (Invitrogen, Waltham, MA, USA). Briefly, the BV2 microglial cells were seeded at 9 × 10^4^ in 24-well plates and incubated overnight at 37 °C. Subsequently, the BV2 microglial cells were treated with 100, 200, and 400 μg/mL of AANCP for 1 h, followed by treatment with 1 μg/mL LPS and incubated in a CO_2_ incubator for 24 h. Next, 100 μL of the coating buffer with capture antibody was dispensed per well, and the microplates were incubated overnight at 4 °C. The coated microplates were washed with phosphate-buffered saline (PBS) (Corning, Corning, NY, USA) containing 0.05% Tween 20 (LPS Solution; Daejeon, Republic of Korea). ELISA/ELISPOT diluent was added to each well (200 μL) and incubated for 1 h. Subsequently, 100 μL media containing AANCP and/or LPS-treated BV2 microglial cells was added to the microplate and incubated for 2 h at room temperature. Each microplate well was treated with 100 μL of detection antibody diluted in ELISA/ELISPOT buffer and incubated for 1 h at room temperature, followed by incubation with 100 μL/well of the ELISA/ELISPOT diluent containing streptavidin-HRP for 30 min at room temperature. The microplates were then incubated with 100 μL of tetramethylbenzidine (TMB) substrate solution per well for 30 min in a dark place. Finally, 100 μL of ELISA stop solution was added to the microplates (Invitrogen). The absorbance was detected at 450 nm using a Varioskan LUX Multimode Microplate Reader (Thermo Fisher Scientific, Waltham, MA, USA).

### 2.7. In Vitro Analysis via qRT-PCR

BV2 microglial cells (5 × 10^4^ cells/well) were seeded in 24-well plates (SPL Life Sciences, Gyeonggi-do, Republic of Korea) for 24 h with 10 μM of Aβ_42_ peptides and 100, 200, and 400 μg/mL of AANCP diluted in the media for 6 h. The control groups were treated with equivalent media. The vehicle groups were exposed to 10 μM of Aβ_42_ peptides only.

The AccuPrep RNA Extraction Kit (Bioneer, Daejeon, Republic of Korea) was used to extract total RNA from the BV2 microglial cells according to the manufacturer’s instructions. RNA was quantified using the SPECTROstar Nano instrument (BMG Labtech, Ortenberg, Germany). The Takara PrimeScript RT Master Mix (Takara, Shiga, Japan) was used to obtain total cDNA from 0.5 μg of total RNA. Prior to the quantitative reverse transcription polymerase chain reaction (qRT-PCR), the cDNA was diluted 10 times in sterile distilled water. Custom primers were created by Bioneer to selectively amplify the genes, including TNF-α, IL-6, IL-1β, and TREM2. 

The forward sequence for TNF-α was TCG TAG CAA ACC AAG TG, while the reverse sequence was ATA TAG CAA ATC GGC TGA CG. The forward sequence for IL-6 was GAG GAT ACC ACT CCC AAC AGA CC, and the reverse sequence was AAG TGC ATC GTT CAT ACA. The forward sequence for IL-1β was GCT ACC TGT GTC TTT CCC GT, and the reverse sequence was CAT CTC GGA GCC TGT AGT GC. The forward sequence of TREM2 was GAC CTC TCC ACC AGT TTC TCC, and the reverse sequence was TCA GAG TGA TGG TGA CGG TTC. The forward sequence of the housekeeping gene GAPDH was TGG CAC AGT CAA GGC TGA GA, and the reverse sequence was CTT CTG AGT GGC AGT GAT GG.

The total volume was 20 μL, containing 10 μL of iQ™ SYBR^®^ Green Supermix (Bio-Rad, Hercules, CA, USA), 1 μL of forward sequence primer, 1 μL of reverse sequence primer, and 8 μL of cDNA. The qRT-PCR data for TNF-α, IL-6, IL-1β, and TREM2 were obtained using a CFX Connect Real-Time PCR Detection System and analyzed using CFX Maestro software 3.0 (Bio-Rad, Hercules, CA, USA). The thermal cycling profiles comprised a single cycle of heating at 95 °C for 3 min, followed by 40 cycles of heating at 95 °C for 15 s, cooling at 59 °C for 30 s, and extension at 72 °C for 30 s. The process concluded with a final cycle of extension at 72 °C for 10 min. The relative expression of each gene was calculated using the ∆Cq technique with GAPDH (glyceraldehyde-3-phosphate dehydrogenase). The quantification cycle (Cq) values were normalized using GAPDH Cq and analyzed using the comparative CT Method 2−∆∆Cq method. 

### 2.8. In Vitro Microglial Phagocytosis Assay

BV2 microglial cells (1 × 10^4^ cells/well) were seeded on an 8-well chamber slide (Ibidi, Gräfelfing, Germany) for 24 h. The AANCP was diluted in conditioned media to concentrations of 100, 200, and 400 μg/mL. The BV2 microglial cells were cultured in various concentrations of AANCP and 500 nM of FAM-Aβ for 1 h. Microglial phagocytosis was quantified by detecting FAM-Aβ within the microglial cells [44]. The cells were incubated with an Iba-1 antibody (1:1000; Abcam, Cambridge, MA, USA) for 2 h at 4 °C in PBST (PBS with 0.1% Tween 20) and 3% bovine serum albumin (BSA). Subsequently, the cells were treated in a solution containing donkey Alexa 594-conjugated anti-goat IgG (1:200; Thermo Fisher Scientific, Waltham, MA, USA) for 1 h at room temperature. DAPI staining and mounting were performed using Fluoroshield™ with DAPI.

### 2.9. Image Acquisition and Analysis

Images were obtained using an LSM 700 microscope (Carl Zeiss AG, Oberkochen, Germany) and processed using the ImageJ software (NIH, Bethesda, MD, USA). The images were captured and examined in a blind manner. Microglia containing FAM-Aβ debris were quantified by enumerating the number of cells exhibiting positive signals for Iba-1 and DAPI. We utilized ImageJ software to measure the FAM-Aβ signal intensities in each group to determine the amount of ingested FAM-Aβ in the BV2 microglial cells.

### 2.10. In Vitro Thioflavin T Assay for the Aggregation of Aβ_42_

ThT assay was performed using Aβ_42_ to examine the effects of AANCP on the aggregation of Aβ. The AANCP at concentrations of 50, 100, 200, 400, and 800 μg/mL was mixed with 25 μM of Aβ_42_ and ThT solution and incubated. In addition, 2 mM morin, which is a highly effective inhibitor of Aβ aggregation, was subjected to incubation at 37 °C for 12-, 24-, 36-, and 48 h as a positive control. Following the incubation period, the ThT fluorescence intensities were measured using a SpectraMax iD3 Multimode Microplate Reader (Molecular Devices, LLC, San Jose, CA, USA) with excitation/emission wavelengths of 440 and 484 nm, respectively. All experiments were conducted in triplicates.

### 2.11. In Vivo Animals and Oral Administration in Mice

Six-week-old female Balb/c mice (Samtako Bio Korea, Osan, Republic of Korea) were acclimatized to the laboratory environment for 7 days and randomly separated into four groups based on an average body weight of 16.7 ± 0.37 g. The four groups were: (1) vehicle-treated mice (n = 4), (2) vehicle-treated mice injected with scopolamine (n = 5), (3) extract of *Ginkgo biloba* (EGB)-treated mice injected with scopolamine (n = 4), and (4) AANCP-treated mice injected scopolamine (n = 4). Table S2 gives experimental groups and concentration of inducer and medication in scopolamine-treated mice for the alleviation of cognitive impairment.

All animals were housed in a laboratory animal room with temperature maintained at 20–25 °C, humidity maintained at 40–45%, and a 12 h light/dark cycle. AANCP and EGB were diluted to 500 and 50 mg/kg, respectively, in saline solution and orally administered daily in the morning for 2 weeks. Scopolamine was dissolved in saline at 2 mg/kg and was injected intraperitoneally 30 min before the behavioral experiments to induce cognitive dysfunction. All groups were administered the same volume of saline and exposed to identical stress conditions for the indicated periods. In addition, all animals were weighed at 3-day intervals to assess animal suffering and abnormalities. All animal experiments adhered to the Principles of the Care and Use of Laboratory Animals (National Institutes of Health publication no. 85-23, revised in 1985) and received approval from the Institutional Animal Care and Use Committee (IACUC) of JBKLAB (JBK-23-07-011).

### 2.12. Y-Maze Test

The Y-maze test utilized a Y-shaped black Plexiglas maze with three 35 cm long × 3 cm wide × 12 cm high arms arranged at 120° angles. Each arm was randomly labeled A, B, or C. Mice were initially placed in the center of the maze to acclimatize for 5 min. Following this, the maze was cleaned with 70% ethanol to remove odors and debris. The mice were then returned to the maze’s center and allowed to explore for 8 min. A Hubble 300 video camera (Screen for You Co., Seoul, Republic of Korea) was placed above the Y-maze to capture the movements of the mice and record them using the Bandicam software (Bandicam Company, Seoul, Republic of Korea). The video images captured during the remaining 6 min, excluding the first 2 min, were analyzed to quantify mice behavior. An alteration was defined as mice entering the three arms sequentially (i.e., CBA, BAC, or ABC) and was considered a correct response. The percentage of spontaneous alterations was calculated using the following formula: [(number of alterations)/(total arm entries−2)] × 100. The total number of arm entries was used to assess the locomotor activity of the mice.

### 2.13. Passive Avoidance Test

The passive avoidance test utilizes the instinct of mice to seek dark places and is conducted to test short-term memory. The box used for the passive avoidance test was divided into a light area and an electronic shock-provided dark area. The mice were allowed to move freely for 300 s to acclimatize to the passive avoidance box. After acclimatization, the mice were returned to their cages, and the passive avoidance box was cleaned with 70% ethanol. The experiment was performed for a total of 2 days. Subsequently, the mice were placed in the illuminated section of the passive avoidance box, and the partition was removed after 30 s. When the mouse entered the dark room, the partition was closed after 5 s, and an electric shock (0.15 mA) was applied for 1 s. After removing the partition, latency times up to 300 s were recorded so that the mice could enter the dark compartment. Twenty-four hours later, the time taken to enter the dark room was measured using the same methodology; however, no electric shock was applied. 

### 2.14. Statistical Analysis

The analyses were performed in a blind manner. Statistical analyses were carried out using GraphPad Prism software (version 9.0; GraphPad, San Diego, CA, USA). The error bars in the graph represent the standard deviations of the data. The statistical significance of differences between groups was assessed using an independent t-test and one-way analysis of variance (ANOVA), followed by Tukey’s post hoc test. Differences were considered statistically significant at *p* < 0.05. 

## 3. Results

### 3.1. Physicochemical Characterization of AANCP

**Figure 1:**
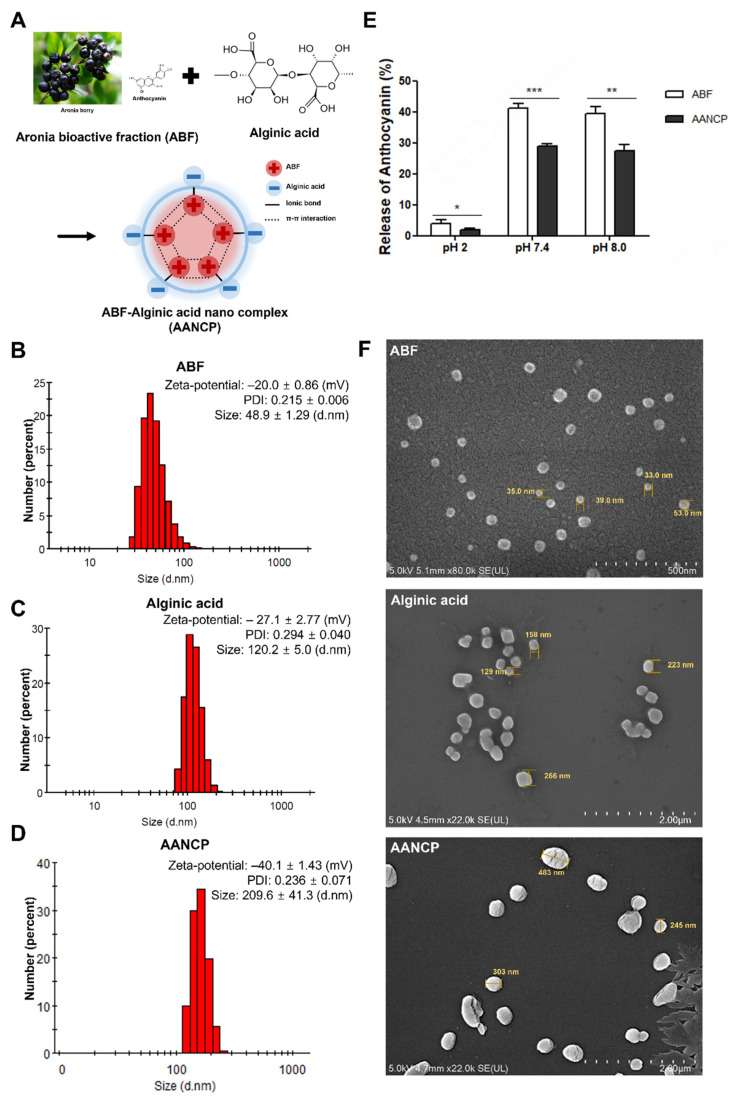
Physicochemical characterization of the ABF-alginic acid nanocomplex. (**A**) Schematic representation of chemical bonding in the AANCP. ABFs are represented by (+) and alginic acids by (−). Solid lines express ionic bonds, and dotted lines represent π-π interactions. Zeta potential, PDI, and distribution of zeta average particle size of (**B**) ABF, (**C**) alginic acid, and (**D**) the AANCP using DLS. (**E**) Anthocyanin release test of ABF and the AANCP according to various pH solutions. (**F**) SEM image of ABF, alginic acid, and AANCP. Statistical analyses were performed using one-way ANOVA followed by Tukey’s test. Significance levels of * *p*-value < 0.05, ** *p*-value < 0.01 and *** *p*-value < 0.001 indicate differences between the ABF-treated group (white bar), and the AANCP-treated group (black bar).

Figure 1 gives a physicochemical characterization of the AANCP. AANCP formed nanoparticles by electrostatically complexing ABF with anionic alginic acid to increase the structural stability and therapeutic benefits of ABF. The AANCP was formed by driving forces, including π-π interactions between molecules of ABF and ionic bonds between ABF and alginic acid (Figure 1A). We measured the zeta-average particle size and zeta potential of ABF, alginic acid, and AANCP using DLS. Zeta potential refers to the potential difference between particles, and PDI exhibits the degree of polydispersity of the particles. Zeta average particle indicates the size of the particles as measured by DLS. The zeta potentials of ABF and alginic acid were observed to be −20.0 ± 0.86 mV and −27.1 ± 2.77 mV, respectively (Figure 1B,C). The zeta-average particle size of the AANCP was measured to be 209.6 ± 41.3 nm, with a zeta potential of ‒40.1 ± 1.43 mV (Figure 1D). 

It is known that chokeberries (Aronia melanocarpa) contain various components such as minerals, vitamins, carbohydrates, amino acids, organic acids, fats, aroma compounds, and especially polyphenols compounds such as anthocyanins, flavonoids, procyanidins, and phenolic acid. Table S1 shows chemical compositions, macro-/ micro-element contents (mg/100 g), and phenolic phytochemicals present in Aronia melanocarpa berries. Although anthocyanin as a main ingredient has a positive charge (approximately +10 mV) because of the overall positive charge of the flavylium cations dominant at low pH conditions, the average zeta potential of ABF was negatively charged. It was reported that the zeta potential of the alginate solution was varied as a function of pH. The sodium alginate solution showed negative zeta potential over the entire pH range, ranging from −33.1 mV at pH 3.0 to −66.7 mV at pH 7.0, attributing to the dissociated carboxyl groups of guluronic and mannuronic acids that are present in the alginate molecule (pKa 3.5) [45]. The zeta potential of alginic acid was almost similar to the reported values. As anthocyanin was electrostatically complexed with negatively charged alginate, the overall zeta potential of AANCP was almost twice further decreased, giving good physical stability.

In addition, the polydispersity index (PDI) values for ABF, alginic acid, and AANCP were 0.215 ± 0.006, 0.294 ± 0.040, and 0.236 ± 0.071, respectively. ABF, alginic acid, and the AANCP were all spherical, but the AANCP exhibited a much larger size because of the formation of an electrostatic complex compared to ABF and alginic acid. The size of the AANCP is attributed to the conjugation of ABF and alginic acid (Figure 1F). Additionally, the release rate of anthocyanins from the AANCP was much slower than that of ABF under all pH conditions (Figure 1E), which may be beneficial for the prolonged treatment of AD-related diseases. In addition, the chemical stability of ABF and AANCP was measured in four different plasmas, and data were presented in Table S3. The C3G concentration was used as a marker for the plasma stability of ABF and AANCP. AANCP gave much higher plasma stability than ABF. The half-life of AANCP was approximately two times longer in human plasma samples, followed by canine, mouse, and rat plasma. These results indicate that the AANCP exhibited greater electrostatic repulsion than ABF, leading to enhanced structural stability and colloidal sizes conducive to absorption in the small intestine by the dynamic equilibrium process.

### 3.2. AANCP Inhibits LPS-Induced Secretion of Pro-Inflammatory Cytokines from Microglia

**Figure 2:**
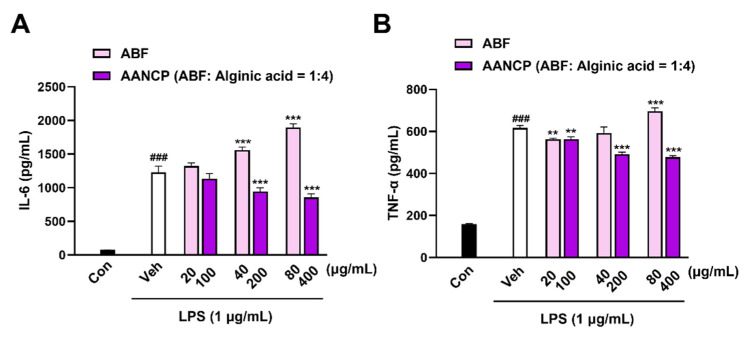
Effect of AANCP on the inhibition of the LPS-induced secretion of pro-inflammatory molecules in BV2 microglial cells. IL-6 and TNF-α levels were determined by ELISA. (**A**) The quantitative analysis shows that AANCP reduced LPS-induced IL-6 release from BV2 microglial cells. (**B**) The quantitative graph shows that AANCP decreased the LPS-induced TNF-α secretion from microglia. The mean ± S.E.M. values were calculated. Statistical analyses were performed using one-way ANOVA followed by Tukey’s test. Differences were significant at ### *p*-value < 0.001 between the control group (black bar) and the vehicle-treated group (white bar). Significance levels of ** *p*-value < 0.01 and *** *p*-value < 0.001 indicate differences between the vehicle-treated group, the ABF-treated group (pink bar), and the AANCP-treated group (purple bar).

We confirmed that no cytotoxicity up to 800 ug/mL was observed in AANCP-treated cells, while ABF-treated cells reduced cell viability above 299 ug/mL concentration using BV2 microglial cells without treating LPS (Figure S1). Subsequently, the secretion of pro-inflammatory molecules released by LPS-treated BV2 microglial cells using ELISA was evaluated to investigate the effect of AANCP on the inflammatory response in comparison to ABF (Figure 2). The secretion levels of IL-6 and TNF-α were significantly elevated in the LPS + vehicle group compared to the control group. Notably, the LPS-induced increased pro-inflammatory cytokines were significantly reduced by AANCP treatment. In particular, IL-6 and TNF-α secretion levels were significantly decreased in the LPS + AANCP group treated with 200 and 400 μg/mL AANCP compared to the LPS + vehicle-treated group (Figure 2A,B). These results indicate that AANCP reduces LPS-induced secretion of pro-inflammatory mediators in BV2 microglial cells.

### 3.3. AANCP Stimulates In Vitro Microglial Polarization from M1 to M2 by Inducing the Expression of TREM2 

**Figure 3:**
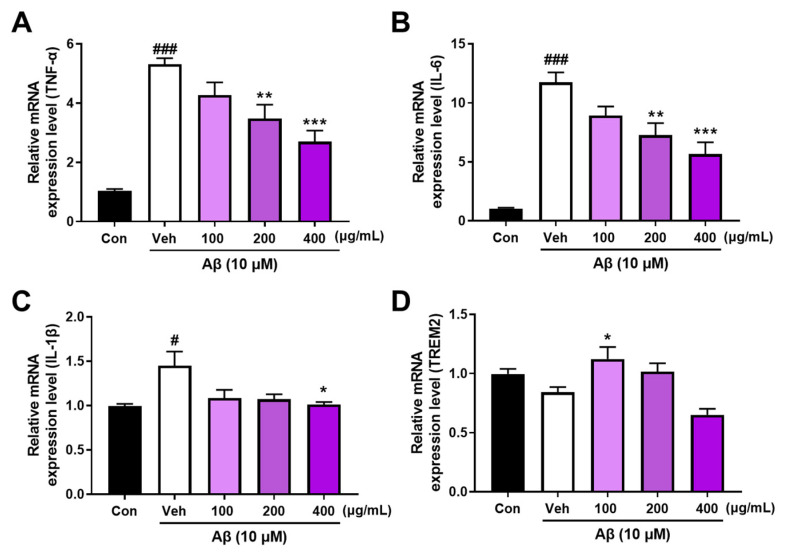
Effect of AANCP on the conversion of the M1 phenotype induced by Aβ to the M2 phenotype in microglial cells. The M1 phenotype was assessed by the expression of cytokines, and the M2 phenotype was examined by the expression of TREM2 in BV2 microglial cells. (**A**–**C**) Quantitative analysis shows that AANCP reduced Aβ_42_-mediated upregulation of M1 markers, including TNF-α, IL-6, and IL-1β, in microglia. (**D**) The quantified graph shows that the AANCP modulated the level of TREM2 mRNA in microglia cells. The mean ± S.E.M. values were calculated. Statistical analyses were performed using one-way ANOVA followed by Tukey’s test. Differences were significant at # *p*-value < 0.05 and ### *p*-value < 0.001 between the control group (black bar) and the vehicle-treated group (white bar). Significance levels of * *p*-value < 0.05, ** *p*-value < 0.01, and *** *p*-value < 0.001 indicate differences between the vehicle-treated group and the AANCP-treated group (purple bar).

We evaluated the expression of M1 cytokines and TREM2 using qRT-PCR to investigate whether AANCP alters the microglial phenotype. Figure 3 shows the effect of AANCP on the conversion of the M1 phenotype induced by Aβ to the M2 phenotype in microglial cells. The mRNA levels of pro-inflammatory cytokines, such as TNF-α, IL-6, and IL-1β, were assessed as markers of the M1 phenotype, while TREM2 expression was evaluated as a marker of the M2 phenotype. The mRNA expression levels of TNF-α, IL-6, and IL-1β were significantly elevated in the Aβ + vehicle-treated group compared to that in the control group (Figure 3A–C). However, treatment with 200 μg/mL of AANCP significantly inhibited the Aβ-mediated up-regulation of TNF-α, IL-6, and IL-1β in BV2 microglial cells, compared to the Aβ + vehicle-treated group. Subsequently, we evaluated the expression levels of TREM2 transcripts in Aβ_42_ and/or AANCP-treated BV2 microglial cells to examine the effect of AANCP on the M2 microglial phenotype. The results showed that the Aβ + vehicle-treated group tended to show diminished expression of TREM2 mRNA compared to that in the control group. However, the Aβ and 100 μg/mL of the AANCP-treated group showed a significant increase compared to the Aβ + vehicle-treated group (Figure 3D). Taken together, these results suggest that AANCP exerts anti-inflammatory effects by modulating the M1/M2 polarization of microglia.

### 3.4. AANCP Activates Phagocytosis in BV2 Microglial Cells

**Figure 4:**
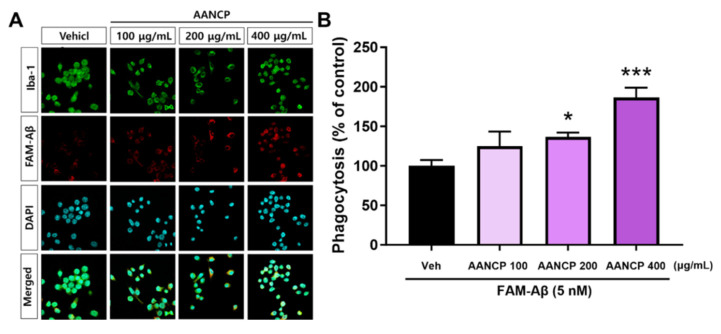
Significant enhancement of AANCP on the microglial phagocytic clearance of Aβ. (**A**) Representative images show immunoreactivity of ionized calcium-binding adaptor molecule 1 (Iba-1; green) and FAM-labeled Aβ_42_ (FAM-Aβ; red) in BV2 microglial cells. DAPI staining was performed to visualize the nucleus (cyan). (**B**) Phagocytosis rates were expressed as a percentage with a counting number of both Aβ_42_- and Iba-1-positive cells per DAPI-positive cells. The mean ± S.E.M. values were calculated. Statistical analyses were performed using one-way ANOVA followed by Tukey’s test. Significance levels of * *p*-value < 0.05 and *** *p*-value < 0.001 indicate differences between the vehicle-treated group and the AANCP-treated group (purple bar).

We hypothesized that the upregulation of TREM2 by AANCP would increase Aβ phagocytosis by microglia. We observed that AANCP elevated the expression of TREM2 in Aβ-treated BV2 microglial cells (Figure 3D). Subsequently, the BV2 microglial cells were exposed to FAM-Aβ, with or without 100, 200, and 400 μg/mL AANCP. Figure 4 gives significant enhancement of AANCP on the microglial phagocytic clearance of Aβ.

Additionally, the microglia were stained with the microglial marker Iba-1, and the number of FAM-Aβ-containing microglia was quantified to investigate the phagocytotic activation by AANCP (Figure 4A). We found that phagocytotic activation by AANCP increased in a concentration-dependent manner, and phagocytosis of Aβ was significantly increased by treatment with 200 μg/mL AANCP compared to the vehicle-treated group. Moreover, in the 400 μg/mL AANCP-treated group, phagocytosis of Aβ was increased by approximately 186% compared to the vehicle-treated group (Figure 4B).

### 3.5. AANCP Inhibits In Vitro Aggregation of Aβ_42_

**Figure 5:**
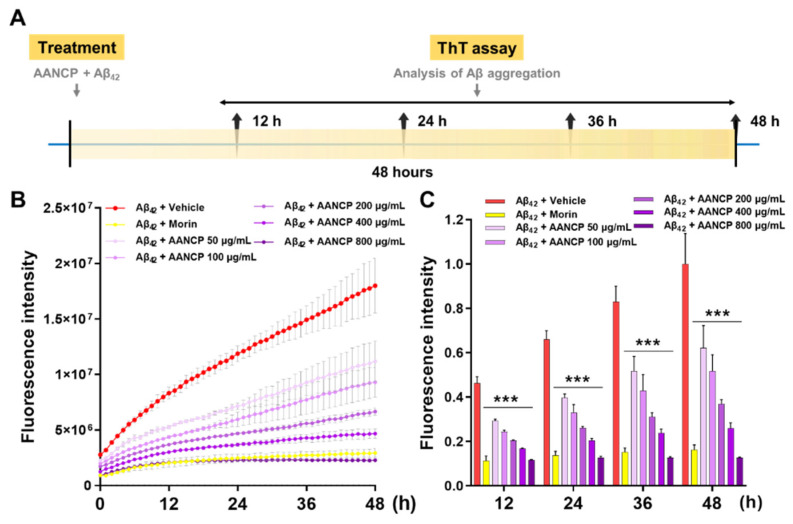
Effect of AANCP on the significant inhibition of Aβ aggregation. (**A**) An outline of the experimental design for treatment and ThT assay, (**B**) ThT fluorescence intensity curves from 0 to 48 h, and (**C**) bar graph at the 12-, 24-, 36-, and 48 h exhibiting the kinetics of Aβ_42_ aggregation, both with and without AANCP. Morin serves as a positive control for the inhibitory activity of Aβ_42_ aggregation. Values are expressed as the mean ± S.E.M. Statistical analyses were performed by one-way ANOVA, followed by Tukey’s test. *** *p*-value < 0.001 indicates significant differences between the Aβ_42_ + vehicle-treated group (red bar) and the Aβ_42_ + AANCP (purple bar) or Aβ_42_ + morin-treated group (yellow bar).

We hypothesized that the newly developed AANCP would also inhibit the aggregation of Aβ_42_. A ThT assay was performed at 12, 24, 36, and 48 h following treatment with 25 μM of Aβ_42_ and 50, 100, 200, 400, and 800 μg/mL of AANCP to determine the inhibitory effect of AANCP on the aggregation of Aβ_42_ (Figure 5A). In the vehicle group, Aβ_42_ aggregation persisted throughout the incubation time. In contrast, the groups treated with various concentrations of AANCP showed a potent decrease in the aggregation of Aβ_42_ compared to the vehicle group in a concentration-dependent manner (Figure 5B). At a concentration of 800 μg/mL, AANCP exerted a more substantial reduction in fluorescence intensity compared to the positive control, 2 mM morin (Figure 5C). These results exhibit that AANCP is an effective compound for inhibiting aggregation of Aβ.

### 3.6. AANCP Ameliorates Cognitive Impairment in Scopolamine-Induced Mice

In preliminary studies, the effect of AANCP on the amelioration of cognitive impairment was compared with ABF in scopolamine-induced Balb/c mice (Figure S2). The body weight and total arm entry representing the locomotor activity of mice were not significantly changed by the administration of ABF and AANCP in Balb/c mice. In contrast, AANCP significantly increased spontaneous alterations (%), indicating the proportion of sequential arm entries in the Y-maze test compared with ABF. Latency time (s) of moving from the light to the dark room before and after the electronic shock trial was also significantly increased in the passive avoidance test as compared with ABF. It was evident that the newly developed AANCP could alleviate cognitive decline in animal models of dementia. 

**Figure 6:**
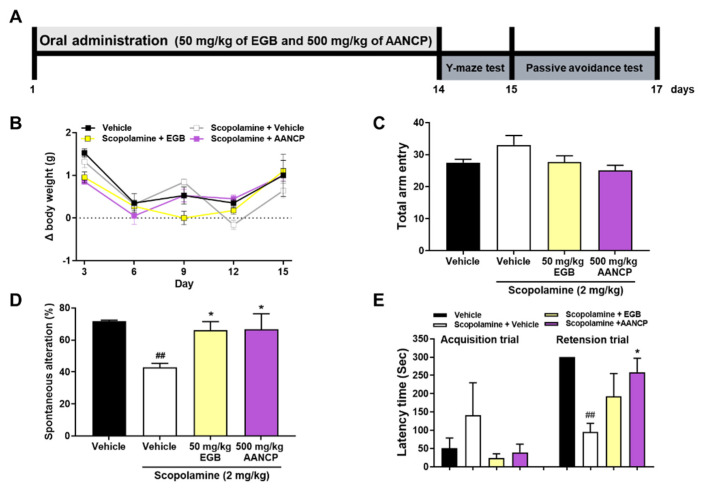
The effect of AANCP on the significant alleviation of cognitive impairment in scopolamine-treated mice. (**A**) Schematic design of vivo experiment. Balb/c mice were orally administered AANCP (500 mg/kg) and EGB (50 mg/kg) daily for 2 weeks. EGB was used as a positive control. Scopolamine (2 mg/kg) was administered intraperitoneally 30 min before the behavioral test to impair cognitive function. (**B**) Changes in body weight by administration of scopolamine, EGB, and AANCP in Balb/c mice. Body weight was measured every 3 days from day 1 to 15. (**C**) Total arm entry, (**D**) spontaneous alterations (%), (**E**) Latency time (s). Values are expressed as the mean ± S.E.M (n = 4 in vehicle-treated Balb/c mice; n = 5 in scopolamine-treated Balb/c mice; n = 4 in scopolamine and EGB-treated Balb/c mice; n = 4 in scopolamine and AANCP-treated Balb/c mice). Statistical analyses were performed by one-way ANOVA, followed by Tukey’s test. ## *p*-value < 0.01 indicates significant differences compared to the vehicle-treated Balb/c mice (black bar) and scopolamine and vehicle-treated Balb/c mice (white bar) and * *p*-value < 0.05 indicates significant differences between the scopolamine and vehicle-treated Balb/c mice and or scopolamine and AANCP-treated Balb/c mice (purple bar).

The effect of AANCP on the significant alleviation of cognitive impairment in scopolamine-treated mice is shown in Figure 6. The Y-maze and passive avoidance tests were used to evaluate the efficacy of AANCP in alleviating cognitive impairment. Scopolamine (2 mg/kg) was administered to each group, except for vehicle-treated Balb/c mice, 30 min before behavioral analysis. EGB, a natural extract that mitigates cognitive decline, was used as a positive control in this study (Figure 6A). We measured changes in body weight to monitor health or adverse reactions (Figure 6B). Body weight was not significantly different between Balb/c mice treated with scopolamine, EGB, or AANP and vehicle-treated Balb/c mice. Subsequently, we conducted the Y-maze and passive avoidance tests to assess the effect of AANCP on cognitive dysfunction. During the Y-maze test, no significant difference was observed in the total arm entry among the groups (Figure 6C). Notably, the percentage of spontaneous alterations in scopolamine-treated Balb/c mice was significantly lower than that in vehicle-treated Balb/c mice. However, a significant increase was observed in the percentage of spontaneous alterations in scopolamine + EBG- or scopolamine + AANCP-treated Balb/c mice compared to scopolamine + vehicle-treated Balb/c mice (Figure 6D). In the passive avoidance test, no significant difference was observed in the latency time between the groups during the acquisition trial before the application of the electronic shock. However, during the retention trial, measured 24 h after the application of electronic shock, scopolamine + vehicle-treated Balb/c mice showed a significant decrease in latency time compared to vehicle-treated Balb/c mice. In contrast, the latency time of EGB- or AANCP-treated Balb/c mice treated with scopolamine was significantly elevated compared to that of scopolamine- and vehicle-treated Balb/c mice (Figure 6E).

## 4. Discussion

To investigate the advanced effects of anthocyanin-rich bioactive fraction (ABF), we combined the low-molecular alginic acid and evaluated stability and efficacy in the present study. First, we measured the structural stability of the newly developed AANCP compared with ABF using zeta potential and morphological analyses. Previous studies have reported that anthocyanins are not structurally stable in vivo [42]. In addition, it has been reported that anthocyanins build electrostatic complexes of anthocyanins through π-π interactions between anthocyanins [46], and cationic anthocyanins are known to form ionic bonds with anionic molecules of other substances. The negatively charged AANCP readily formed homogeneous nanoparticles with a consistent polydispersity index (PDIs, Figure 1F). PDI serves as an indicator of particle size uniformity, with lower values suggesting a more homogeneous particle size distribution. It was known that the size range of the nanocomplexes suitable for penetrating the epithelial cells in the small intestine is −50–500 nm. Zeta potential, termed electrokinetic potential, is the slipping/shear plane of a colloid particle moving under an electric field, and it reflects a potential gap between the electric double layer of particle and the layer of dispersant around them. Also, it is well known that the larger the zeta potential, the more stable the substance [47]. AANCP showed a bigger zeta potential compared to each substance (Figure 1B–D), and the data indicate that AANCP is more stable than ABF, which was in accordance with another study [48]. Our pH and plasma stability data indicated that C3G in AANCP was maintained for a longer duration compared to ABF (Figure 1E and Table S3). C3G, a representative marker and active compound of Aronia species [49] and the most frequently observed molecule in human intervention studies [50], is utilized as an indicator to assess the plasma stability of AANCP.

Previous studies have reported that anthocyanins inhibit pro-inflammatory cytokines, such as TNF-α, IL-6, and IL-1β [51,52,53]. Based on these reports, we evaluated the effectiveness of the newly developed AANCP in reducing pro-inflammatory cytokine levels and alleviating neuroinflammation. We hypothesized that AANCP would break the vicious cycle of Aβ pathology and neuroinflammation by modulating neuroinflammation and inhibiting Aβ aggregation. In addition, alginic acid, in combination with ABF, has been reported to interact with acetylcholinesterase, an effective target for alleviating cognitive impairment. It was also reported that the expression of pro-inflammatory cytokines and TREM2 can distinguish the M1/M2 phenotype of microglia [13,20,54]. TREM2, a transmembrane receptor on microglia, is known to induce microglial phagocytosis of Aβ [55,56]. The upregulation of TREM2 observed during the transition of microglia from the M1 to the M2 phenotype indicates that TREM2 may serve as a potential marker for identifying the M2 phenotype of microglia [57]. C3G, one of the main bioactive components of anthocyanin, regulates the M1/M2 polarization of microglia treated with Aβ through the up-regulation of TREM2 [58].

AANCP significantly inhibited the secretion of pro-inflammatory cytokines, such as TNF-α and IL-6, in LPS-treated BV2 microglial cells. However, In the treatment group receiving only ABF, the secretion of IL-6 induced by LPS exhibited a dose-dependent increase (Figure 2). These results are consistent with reports that chlorogenic acid and cyanidin, components of Aronia berry extract, increase LPS-induced IL-6 secretion in human astrocytes. Natural extracts consist of multiple substances that can complement or counteract each other [59]. In this study, we suggest that the developed AACNP can complement these effects of ABF and enhance its anti-inflammatory activity. AANCP reduced the expression of pro-inflammatory cytokines and elevated the expression of TREM2 in Aβ-treated BV2 microglial cells, suggesting that the AANCP induced the transition of the microglial cells to a TREM2-related M2 phenotype (Figure 3). Moreover, the M2 phenotype, which was switched by AANCP, significantly elevated Aβ microglial phagocytosis (Figure 4). Targeting both neuroinflammation and Aβ pathology may mitigate AD progression because neuroinflammation is closely associated with Aβ pathology in the AD brain [60,61,62,63]. In addition, previous studies have reported that polyphenols, cyanidin-3-galactoside (Cy3Gal), and anthocyanin inhibit the aggregation of Aβ [64,65]. These results suggest that AANCP exhibits significant potential to enhance the phagocytic process, resulting in the clearance of Aβ.

Finally, we found that AANCP significantly inhibited the aggregation of Aβ and alleviated the cognitive impairment in mice induced by scopolamine (Figure 5 and Figure 6). These results indicate that AANCP is an effective agent for ameliorating cognitive decline in an animal model of dementia. The main clinical symptom of AD is cognitive decline [66]. Previous reports have shown that anthocyanins and C3G alleviate cognitive impairment in animal models of AD [67,68,69], as well as in healthy older adults and patients with mild cognitive impairment [70,71]. Also, anthocyanins are promising therapeutic agents for the treatment of AD as they inhibit Aβ aggregation and the formation of neurofibrillary tangles, as well as alleviate mitochondrial dysfunction and synaptic dysfunction [39]. Previous reports indicate that anthocyanins accumulate in epithelial cells and brain parenchyma, including the cortex, striatum, and hippocampus, and their metabolites are observed in the brain tissue of various animal models [72,73,74,75]. Thus, the anthocyanins may mitigate cognitive impairment by direct passage through the BBB or indirectly through the gut–brain axis [76]. Further study needs to confirm whether the pharmacological actions of AANCP are due to the passage of anthocyanins through the BBB. 

Microglia are immune cells in the brain that undergo changes in their cellular characteristics, known as transitions between the M0, M1, and M2 stages, in response to the environment [77,78]. Upon first exposure to Aβ or LPS, microglia transform into the M2 phenotype [79,80,81]. Consequently, they release anti-inflammatory cytokines, including IL-10, TGF-β, and IGF-1, to regulate inflammation [82,83]. However, under chronic exposure, microglia transform into the M1 phenotype [84,85]. Similarly, we observed an increase in pro-inflammatory cytokines in Aβ-treated BV2 microglial cells. Thus, we believed that Aβ- and high concentrations of AANCP-treated BV2 microglial cells show the transition of microglia to the M1 phenotype. We observed that the expression of TREM2, a hallmark of the M2 phenotype, decreased with increasing AANCP concentrations (Figure 3). Therefore, we believe that the decreased expression of TREM2 is associated with changes in microglial phenotype depending on the time of Aβ and AANCP treatment. Consequently, we treated BV2 microglial cells with Aβ and AANCP for 1 and 6 h, respectively, to examine the effect of AANCP on Aβ phagocytosis and expression of pro-inflammatory cytokines. In the acute phase (1 h), a high concentration of AANCP is expected to induce microglia into the M2 phenotype and rapidly eliminate Aβ, while in the chronic phase (6 h), microglia are expected to convert to the M1 phenotype. As shown in Figure 3, high-dose AANCP converted microglia to the M2 phenotype in the early phase; however, over time, the microglial phenotype changed from M2 to M1. Thus, the expression of TREM2, an M2 marker, was reduced. In the present study, the concentration of AANCP may affect the phagocytic capacity of the microglia, resulting in a transition back to the M0 state from the M1 and M2 phenotypes.

AD is strongly associated with neuroinflammation and Aβ pathology [86]. Aβ is a stimulator of neuroinflammation, resulting in inducing the release of pro-inflammatory cytokines and gliosis in AD brains [87]. On the contrary, neuroinflammation induces the production of Aβ, leading to increased Aβ accumulation. It has been reported that inhibition of Toll-like receptor 2 receptors expressed on microglia significantly increases Aβ deposition and impairs cognitive function in mice [88,89]. Moreover, the deletion of the NLRP3 gene in APP/PS1 mice reduces Aβ deposition and alleviates cognitive impairment [90]. These pieces of evidence suggest a vicious circle between neuroinflammation and Aβ pathology, exacerbating the progression of AD [91]. Indeed, there is growing interest in neuroinflammation as a target for the development of AD drugs. Approximately 16% of all drugs in clinical trials for AD target neuroinflammation [92], with Xpro1595 [93] and AL002 [94,95] representing some examples. Neuroinflammation not only forms a vicious circle with Aβ pathology but may also serve as a biomarker of AD. Our data suggest that AANCP attenuates the progression of neurodegeneration by breaking the vicious cycle between neuroinflammation and neurodegeneration, which may lead to the alleviation of cognitive impairment. 

We confirmed that AANCP is superior to ABF only via the regulation of protein levels of inflammatory cytokines in LPS-stimulated BV2 microglial cells. Furthermore, these effects resulted in an improvement in cognitive function in scopolamine-induced dementia mice. Therefore, subsequent investigations focused on assessing the AANCP efficacy. Further studies in other animal models of AD are also required to investigate whether the anti-inflammatory, Aβ scavenging, and anti-aggregation properties of AANCP attenuate neuronal death.

**Figure 7:**
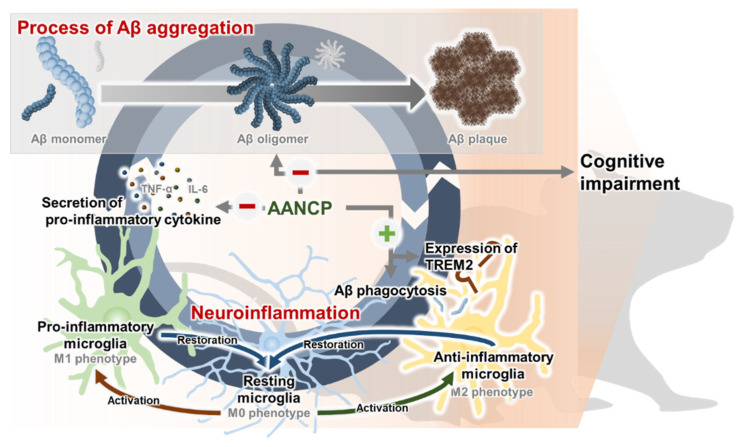
Schematic diagram of the effects of AANCP on neuroinflammation, Aβ aggregation, and cognitive impairment in AD and dementia. The aggregation of Aβ and M1 markers, such as TNF-α and IL-6, form a vicious circle that exacerbates the progression of AD. The pro-inflammatory cytokines promote the aggregation of Aβ, which in turn induces the release of inflammatory molecules. During pathological conditions caused by Aβ, resting microglia are activated into pro-inflammatory or anti-inflammatory microglia. However, upon return to physiological conditions via the removal of Aβ, M1- or M2-activated microglia are restored to resting microglia. AANCP promotes the transition of microglia to the anti-inflammatory phenotype and inhibits the aggregation of Aβ. Consequently, AANCP inhibits Aβ aggregation, reduces neuroinflammation, and improves cognitive impairment in AD. Stimulation is indicated by (+) arrows. Inhibition is indicated by (−) arrows.

Collectively, these results suggested that highly stabilized AANCP could be utilized for the treatment of neuroinflammation, a promising AD target. Figure 7 shows the mechanistic understanding of AANCP to reduce neuroinflammation, inhibit Aβ aggregation, and alleviate cognitive impairment in AD.

## 5. Conclusions

An AANCP was successfully developed to increase the chemical and structural stability of anthocyanin via electrostatic interaction between anthocyanins and alginic acid. The AANCP readily formed nanoparticles and had negatively charged zeta potential. The release rate of anthocyanin from AANCP was slowly liberated in three different media as compared with free anthocyanin due to the molecular interaction. The AANCP readily inhibited the hydration of anthocyanins and increased their intestinal absorption. Notably, AANCP decreased the expression of pro-inflammatory cytokines and increased Aβ phagocytosis through a phenotypic shift in microglia. AANCP also inhibited the aggregation of Aβ and ameliorated cognitive impairment in scopolamine-induced mouse models of dementia. The switching of microglia from the M1 to M2 phenotype was also stimulated. It was evident that AANCP could provide therapeutic benefits associated with AD or neuroinflammation diseases.

## Data Availability

All the data and materials used in this study are available from the corresponding author upon request.

## Supplementary Materials

The following supporting information can be downloaded at https://www.mdpi.com/article/10.3390/pharmaceutics17010013/s1, Table S1: Chemical compositions, macro-/ micro-element contents (mg/100 g), and phenolic phytochemicals present in Aronia melanocarpa berries. Table S2: Experimental groups and concentration of inducer and medication in scopolamine-treated mice for the alleviation of cognitive impairment. Table S3: The chemical stability of free ABF and AANCP in various species plasmas by determining the amount of C3G remaining; Figure S1. Comparison of the effects of ABF and AANCP on BV2 microglial cell viability; Figure S2. Comparison of ABF and AANCP in scopolamine-induced mice.

## Abbreviations

Alzheimer’s disease, AD; Amyloid beta, Aβ; Lipopolysaccharide, LPS; Interleukin 1β, IL-1β; Tumor Necrosis Factor-α, TNF-α; Triggering receptor expressed on myeloid cell 2, TREM2; Food and Drug Administration, FDA; Nuclear factor kappa-light-chain-enhancer of activated B cells, NF-κB; Anthocyanin-alginic acid nanocomplex, AANCP; 5-Carboxyfluorescein-labeled Aβ_1–42_, FAM-Aβ; 4, 6-diamidino-2-phenylindole Fluoroshield, DAPI; Ionized calcium-binding adapter molecule 1, Iba-1; Dynamic light scattering, DLS; Field-emission scanning electron microscope, FE-SEM; Cyanidin-3-O-glucoside, C3G; Cyanidin-3-galactoside, Cy3Gal; Liquid chromatography–mass spectrometry/mass spectrometry, LC-MS/MS; Dulbecco’s Modified Eagle Medium/F12, DMEM/F12; Water-soluble tetrazolium 1, WST-1; enzyme-linked immunosorbent assay, ELISA; phosphate buffered saline, PBS; Tetramethylbenzidine, TMB; quantitative reverse transcription polymerase chain reaction, qRT-PCR; glyceraldehyde-3-phosphate dehydrogenase, GAPDH; quantification cycle, Cq; serum albumin, BSA; extract of Ginkgo biloba, EGB; Institutional Animal Care and Use Committee, IACUC; One-way analysis of variance, ANOVA; polydispersity index, PDI; Thioflavin-T, ThT; Anthocyanin-fucoidan nanocomplex, AFNC; NLR family pyrin domain containing 3, NLRP3.
