# Supplementary material for: Investigation of Novel Aronia Bioactive Fraction-Alginic Acid Nanocomplex on the Enhanced Modulation of Neuroinflammation and Inhibition of Aβ Aggregation
Source: Pharmaceutics. 2024 Dec 25;17(1):13. doi: 10.3390/pharmaceutics17010013 (PMC11769017; doi:10.3390/pharmaceutics17010013)
Supplement: Supplementary file 1 [file pharmaceutics-17-00013-s001.zip › pharmaceutics-3350282-supplementary.pdf]

pharmaceutics

MDPI

Supporting Information

Investigation of Novel Aronia Bioactive Fraction-Alginic Acid Nanocomplex on the Enhanced Modulation of Neuroinflammation and Inhibition of Aβ Aggregation

Bong-Keun Jang [1,2], Soo Jung Shin [3], Hyun Ha Park [3], Vijay Kumar [3], Yong Ho Park [3], Jeom-Yong Kim [2,4], Hye-Yeon Kang [2], Sunyoung Park [2], Youngsun Kwon [2], Sang-Eun Shin [2], Minho Moon [3,5*] and Beom-Jin Lee [1,6*]

Table S1. Chemical compositions, element contents (mg/100 g) and phenolic constituents present in aronia melanocarpa berries (Tomislav Jurendi and Mario Šˇcetar. Aronia melanocarpa Products and By-Products for Health and Nutrition: A Review, **Antioxidants,** 2021, 10, 1052. https://doi.org/10.3390/antiox10071052).

Chemical Composition	Barries g/kg
Dry matter%	15-31
pH	3.3-3.7
Titratable acidity (g citric acid per 100 g)	0.5-1
Total sugar	68-158
Glucose	11-40
Fructose	14-42
Sorbitol	44-76
Total Polyphenols (Chromatographic method)	79
Fiber	56
Minerals	4-6
Fat	1.4
Proteins	7
Amygdalin mg/100 g	20
**Elements**	**Berries[25]**
K	271-498
Ca	60-117
P	24-96
Mg	16-58
Na	1-2
Zn	1.4-0.8
Fe	0.9-1.4
Se	0.02
Cu	0.08-0.2
Mo	0.002
Cr	0.05
Mn	0.5-1.8
Si	0.2-0.6
Ni	0.01-0.07
B	0.3-1.4
V	0.04-0.2
Pb	0.005-0.009
Cd	0.02-0.004
As	0.03-0.04

**Phenolic Constituents**	**Fruit Mg/100g DW[8,9]**	**Fruit mg/100g FW[6,13,29]**
Flavan-3-ol		
(−)-Epicatechin	15	32
Procyanidins	5182	1646
Degree polymerization (DP)	23	59
Anthocyanins		
Cyanidin-3-O-galactoside	19-1282	417-636
Cyanidin-3-O-glucoside	0.3-42	8-27
Cyanidin-3-O-arabinoside	6.2-582	129-299
Cyanidin-3-O-xyloside	53	29-38
Phenolic acids		
Chlorogenic acid	16-302	72-111
Neochlorogenic acid	92-291	59-100
3,4-Dihydroxyphenylacetic acid	4-26	
Protocatechuic acid	0.4-31	
Rosmarinic acid	9-18	
Flavonols		
Quercetin	12-44	7.1
Quercetin-3-O-galactoside	37	7-13
Quercetin-3-O-glucoside	22	4
Quercetin-3-O-rutinoside	15	4
Quercetin-3-O-rhamnoside		
Quercetin-3-O-vicianoside		3-5
Quercetin-3-O-robinobioside		1-5
Quercetin derivates unidentified	27	
Kaempferol		0,5
Flavanon		
Eriodictyol-7-O-glucuronide		24
	DW: dry weight; FW: fresh weight	

Table S2. Experimental groups and concentration of inducer and medication in scopolamine-treated mice for the alleviation of cognitive impairment.

**Groups**	**Inducer of cognitive decline (i.p.)**	**Treatment (p.o.)**
Control	Saline (vehicle)	Saline (vehicle)
Vehicle-treated group	Scopolamine (2 mg/kg)	Saline (vehicle)
EGB-treated group	Scopolamine (2 mg/kg)	EGB (50 mg/kg)
AANCP-treated group	Scopolamine (2 mg/kg)	AANCP (500 mg/kg)
Abbreviation: EGB; extract of *ginkgo biloba*, AANCP; Anthocyanin-alginic acid nanocomplex		

Table S3. The chemical stability of free ABF and AANCP in various species plasmas by determining the amount of C3G remaining.

**Time (min)**	**Amount of C3G remaining in plasmas (%)**							
	**Human**		**Canine**		Rat		Mouse	
	**ABF**	**AANCP**	**ABF**	AANCP	ABF	AANCP	ABF	AANCP
0	100.0 ± 0.030	100.0 ± 0.119	100.0 ± 0.079	100.0 ± 0.066	100.0 ± 0.001	100.0 ± 0.001	100.0 ± 0.021	100.0 ± 0.022
20	89.4 ± 0.008	95.8 ± 0.006**	79.1 ± 0.005	90.2 ± 0.032**	79.4 ± 0.008	78.0 ± 0.006	80.7 ± 0.048	81.7 ± 0.022

40	81.7 ± 0.061	92.6 ± 0.057	72.3 ± 0.025	92.6 ± 0.057**	72.6 ± 0.021	65.0 ± 0.019	68.0 ± 0.000	73.4 ± 0.017**
60	77.9 ± 0.023	90.2 ± 0.074*	68.1 ± 0.035	79.1 ± 0.009**	57.7 ± 0.004*	55.1 ± 0.001	64.3 ± 0.014	68.7 ± 0.004*
120	59.3 ± 0.002	77.1 ± 0.004***	54.0 ± 0.022	61.7 ± 0.047*	49.3 ± 0.022	52.5 ± 0.016	41.8 ± 0.019	46.9 ± 0.018*
Half Life (t1/2) min	163.79	323.23	147.61	176.42	121.13	136.24	99.50	115.47

*p-value < 0.05, ** p-value < 0.01, ***p-value < 0.001 indicate significant differences between the ABF and AANCP

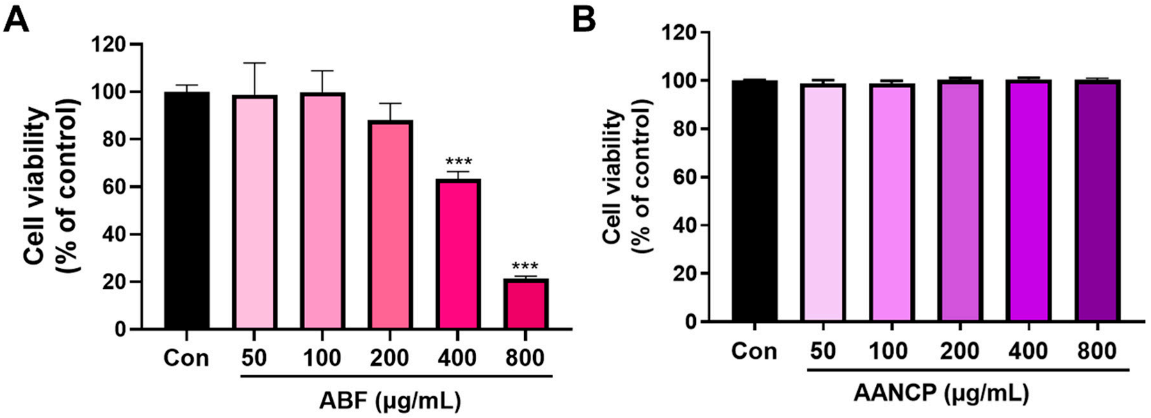

Figure S1. Comparison of the effects of ABF and AANCP on BV2 microglial cell viability without treating LPS. BV2 microglial cell viability was measured following incubation with (A) ABF and (B) AANCP at indicated concentrations for 24 h. The mean ± S.E.M. values were calculated. Statistical analyses were performed using one-way ANOVA followed by Tukey's test. Significance levels of ***p-value < 0.001 indicate differences between the control group (black bar) and the ABF-treated group (red bar), AANCP-treated group (purple bar).

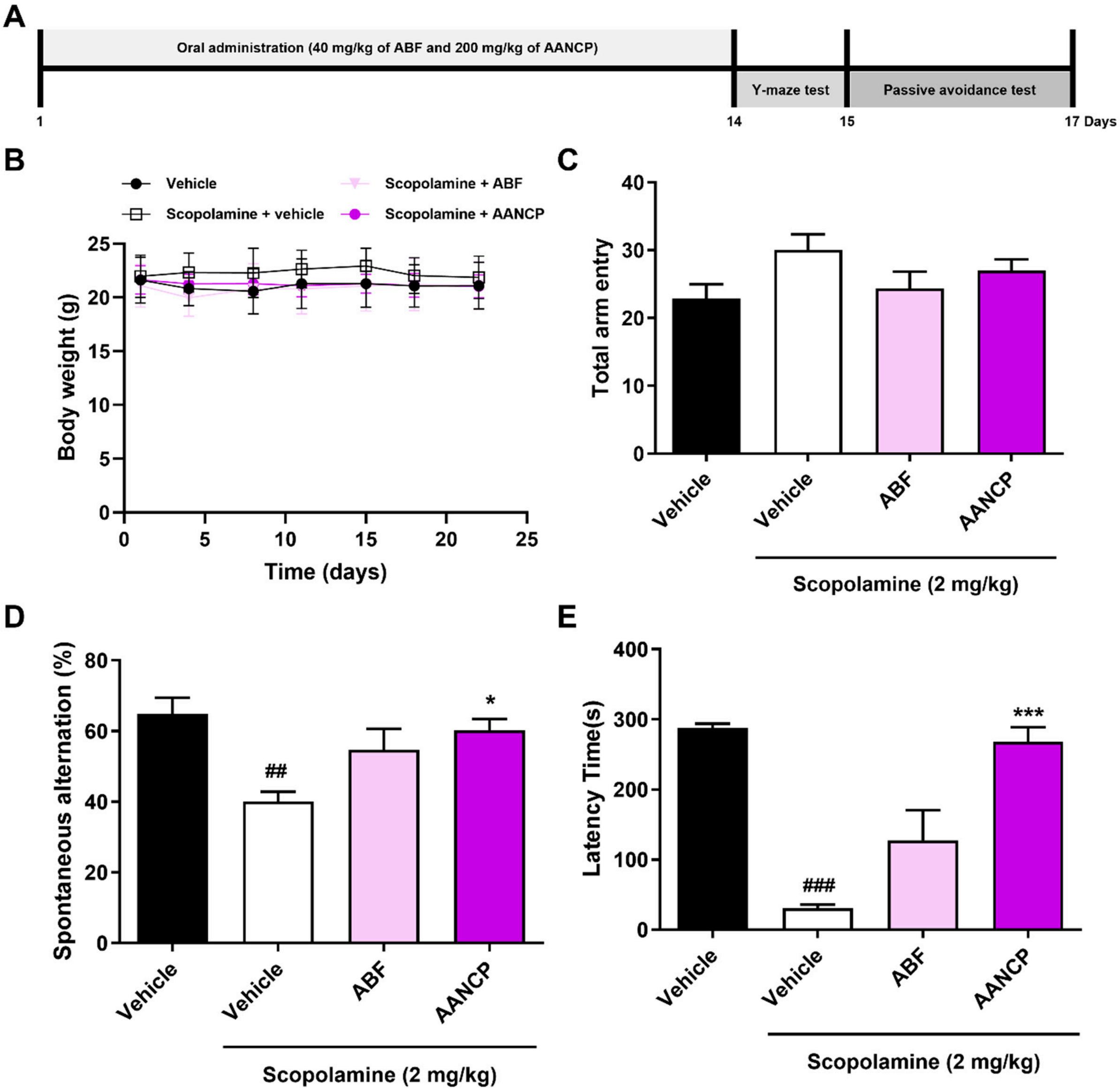

Figure S2. Comparison of ABF and AANCP in scopolamine-induced mice. (A) Overview of the progression of the experiment in vivo. Balb/c mice were orally administered AANCP (200 mg/kg) and ABF (40 mg/kg) daily for 2 weeks. On the final day, scopolamine (2 mg/kg) was administered intraperitoneally 30 min before the behavioral test to impair cognitive function. (B) Body weight was measured every 3 days from day 1 – 15. (C) Total arm entry, (D) Spontaneous alterations (%), (E) Latency time of moving from the light to the dark room before electronic shock trial and after electronic shock trial in the passive avoidance test. Values are expressed as the mean ± S.E.M (n = 5 in vehicle-treated Balb/c mice; n = 5 in scopolamine-treated Balb/c mice; n = 5 in scopolamine and ABF-treated Balb/c mice; n = 5 in scopolamine and AANCP-treated Balb/c mice). Statistical analyses were performed by one-way ANOVA, followed by Tukey's test. ##p-value < 0.01, ###p-value < 0.001 indicates significant differences compared to the vehicle-treated Balb/c mice (black bar) and scopolamine and vehicle-treated Balb/c mice (white bar) and *p-value < 0.05, ***p-value < 0.001 indicates significant differences between the scopolamine and vehicle-treated Balb/c mice and or scopolamine, ABF-treated Balb/c mice (pink bar) and AANCP-treated Balb/c mice (purple bar).
